# Under the Weather: The Meteorological Effect on Orthopaedic Trauma in Hertfordshire

**DOI:** 10.7759/cureus.31146

**Published:** 2022-11-06

**Authors:** Alexander Jaques, John Hanrahan, Sumaya Islam, Rajesh Sofat, Martinique Vella-Baldacchino

**Affiliations:** 1 Trauma and Orthopaedics, Lister Hospital, Stevenage, GBR; 2 Orthopaedics and Traumatology, St Mary's Hospital, London, GBR

**Keywords:** winter months, ankle fractures, theatre efficiency, weather conditions, neck of femur fractures

## Abstract

Background

Effective and efficient use of operating theatres is essential to the smooth running of a trauma service. The paper aims to understand the effect of meteorological factors on the number of referrals and volume and nature of trauma operating cases within our local area.

Methods

Trauma data over two seasons were analysed in our database, a digital clinical platform that coordinates all admissions and trauma theatre activity. Data consisted of the number of referrals per day, patient age, mechanism of injury, and type of orthopaedic injury. Weather data were gathered from ‘Weather Underground’, https://www.wunderground.com/history, which records daily weather observations, located 12 miles away from our trauma unit.

Results

During the study period's last two seasons, 1160 consultations were analysed and 779 required operative intervention. The neck of femur fractures and ankle trauma were the two most common causes of trauma, accounting for 27% and 15%, respectively. The neck of femur fracture pathologies were not significantly correlated with any meteorological factor studied. On the contrary, ankle trauma was the only injury significantly correlating with temperature (p < 0.03) and dew point (p < 0.04). The most common mechanism of trauma was a ground-level fall (n = 590) whilst the least common was a motor vehicle accident (n = 39). Analysing the effect of weather and its effect on the age group of presentation, temperature (p < 0.01), sunlight (p < 0.002), and dew point (p < 0.03) were all significantly correlated with trauma in patients aged younger than 21 years of age.

Conclusion

The weather has no effect on the neck of femur fractures, the most common trauma pathology treated in our department. In all seasons, allocated specific trauma lists for the latter should be arranged irrelevant of the weather conditions. A strong correlation was identified between ankle trauma and weather. We identified that Tuesdays and Fridays received the highest referral rate and peaked between the months of October-November. These data lay the groundwork for local clinical directors to shape the future on-call trauma service.

## Introduction

The precision of meteorological data recording has dramatically improved over the past decade; however, its use in capacity planning within the National Health Service (NHS) is limited. Apart from the NHS, changes in weather affect the commercial sector, such as the retail industry, as it influences consumer decision-making. Stulec (2006) recommends retail outlets use weather data to achieve planned sales targets such as lowering product prices in relation to adverse weather conditions [1]. Equally, trends for orthopaedic trauma workloads are known to follow seasonal variation (temperature, pressure, humidity, rainfall, air circulation, etc.), namely, snowfall and rainfall appear to be linked to a high volume of trauma admissions with a lower tendency for motor vehicle accidents due to increased driver cautiousness [2,3].

Jones (2009) echoed the need to record the effect of adverse weather on trauma workload within a local area to reliably predict capacity [3]. Lister Hospital, Stevenage lies 92 metres above sea level, and the climate in Stevenage is classified as warm and temperate according to the Koppen and Geiger classification, with an average annual temperature of 9.8 degrees centigrade and 632 mm of rainfall [4]. The 730-bed district general hospital based in Hertfordshire, United Kingdom, provides care to a population of around 600,000.

Running operating theatres is one of the most expensive resources and thus must be used efficiently [5]. Unpredictable numbers of admissions put significant strain on operating room and theatre staff availability. This can result in delays in definitive surgical management and subsequently can result in difficulty in delivering timely patient care [6].

Departmental directors and the trauma admission team share responsibility for making the system work and planning enough capacity to cope with the demands of the service, particularly with severe weather variations known to exacerbate the stress on health systems.

The paper aims to improve our understanding on how to plan adequate trauma capacity suited to the needs of emergency trauma care in relation to the meteorological variation within our local area, analysing the influence of weather-related injuries not only on that day but on subsequent days. The paper hopes to use these data to predict local hospital bed planning and trauma theatre capacity.

This article was previously presented in poster format at the National Research Collaborative Meeting (NRCM) on 1st December 2020 and the abstract was subsequently published in the British Journal of Surgery (BJS) Open on 8th April 2021 [7].

## Materials and methods

Trauma data from 23 September 2019 to 20 March 2020 were collected from our institutional database (eTrauma), a digital clinical platform that coordinates all trauma admissions and trauma theatre activity.

For our 180-day study period, data included the number of referrals per day, patient age, and mechanism of injury (motor vehicle crash, ground-level fall, fall from height (FFH), and other blunt mechanisms). Orthopaedic injuries were classified as foot (talus, midfoot, metatarsal, phalanx, and calcaneus), ankle (pilon and ankle), hip (all neck of femur fractures), periprosthetic, pelvis (acetabulum and pelvic ring), femur (shaft and distal femur), tibia (plateau and shaft), spine, chest trauma, clavicle, humerus, forearm (both radius and ulna and olecranon), and distal radius. If a patient had multiple injuries, this was counted as one referral but each injury was tabulated independently for categorical analysis.

At our institution, hand injuries are managed by plastic surgery. Spinal and pelvic traumas are managed at our closest major trauma centre, unless deemed appropriate to be managed locally following discussion with the latter. The number of trauma theatre surgeries was obtained from our electronic database (PathpointTM eTrauma, Open Medical Ltd, London, UK) [8].

Weather data were gathered from ‘Weather Underground’, https://www.wunderground.com/history, which records daily weather observations from Luton Airport Weather Station located 12 miles away from our trauma unit. The weather variables included in this dataset were average temperature (°C), dew point (°C), humidity (%), and maximum wind speed (mph), and hours of daylight were recorded for each day in the study period [9].

Data were recorded into a Microsoft Excel spreadsheet (Microsoft Corporation, Redmond, WA) and a Poisson regression model was performed to understand the effect of meteorological variables on trauma. The study sought to observe the effect of weather on the number of referrals, the volume of trauma operating cases, and bed occupancy within the orthopaedic department.

## Results

**Figure 1:**
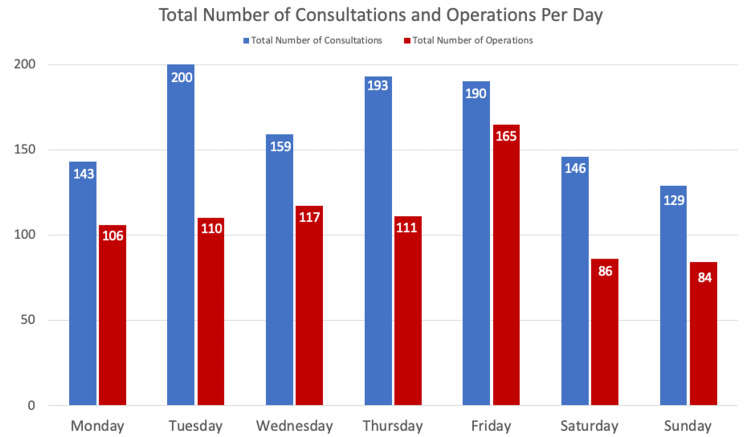
Total number of consultations and operations per day

**Figure 2:**
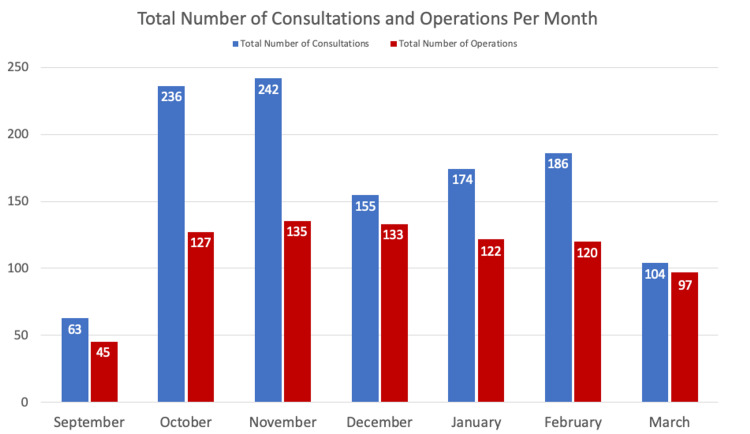
Total number of consultations and operations per month Trauma data between 23 September 2019 and 20 March 2020 were collected from our institutional trauma database (eTrauma).

The study period between the two seasons (autumn and winter 2019-2020) consisted of 180 days. Lister Hospital, Stevenage received 1160 consultations with 779 requiring operative intervention. There were on average seven consultations per day, with a range of 0-16 per day, the highest number of consultations took place on a Tuesday and the least on a Sunday (Figures 1, 2). During the full month study period, the trauma and orthopaedic department received the least number of referrals in December and the highest number in November (Figure 2).

The average number of operative interventions was four per day, with a range of 1-11 interventions. Friday was the day recording the highest number of surgeries and the weekend recorded the least. An increasing trend of operative interventions between September 2019 and peaking in November 2019 was recorded subsequently decreasing until the end of the study period (Table 1).

**Table 1:** Total number of consultations and operations per season

	Autumn	Winter
Total number of consultations	648	512
Average number of consultations (min-max)	7 (1-16)	6 (1-14)
Total number of operations	395	384
Average number of operations (min-max)	4 (1-11)	4 (1-9)

A total of 802 injuries were reviewed or operated on by the trauma and orthopaedic department based in Lister Hospital. The two most common pathologies were the neck of femur and ankle fractures whilst the least common was patella fractures. The most common cause of trauma was a ground-level fall (n = 590) whilst the least common was a motor vehicle accident (n = 39). The most common age group treated by the department were those aged greater than 65 with the least common aged between 22 and 34 years of age (Table 2).

**Table 2:** Poisson regression model analysing the effect of meteorological measures on age group, mechanism of injury, and type of injury Statistical significance was determined with a p-value < 0.05 and results were marked with an asterisk (*).

	Total number	Temperature (p-value)		Sunlight		Dew point	
	N	P-value	RR (%)	P-value	RR (%)	P-value	RR (%)
Age group							
Age groups <21	124	0.01*	0.929	0.002*	1.011	0.03*	0.998
Age groups 22-34	77	0.68	0.990	0.55	1.978	0.55	0.579
Age groups 35-44	66	0.03*	0.920	0.48	1.027	0.07	0.621
Age groups 45-54	105	0.70	0.990	0.75	2.011	0.75	0.473
Age groups 55-64	130	0.38	0.960	0.20	1.469	0.60	0.822
Age groups 65+	502	0.47	0.990	0.47	1.593	0.34	0.625
Mechanism of injury							
Mechanism 1 - motor vehicle crash (MVC)	39	0.48	0.96	0.69	1	0.70	0.98
Mechanism 2 - ground level fall - including fall from a bike	590	0.65	1	0.78	1	0.55	1
Mechanism 3 - fall from height (FFH) including falls from stairs	121	0.41	1.03	0.04*	1	0.50	1.02
Mechanism 4 - other blunt mechanisms (penetrating injury)	37	0.20	1.09	0.29	1	0.18	1.09
Type of injury							
Foot (talus, midfoot, metatarsal, phalanx, calcaneum)	20	0.90	1.04	0.49	1	0.71	0.97
Ankle (pilon, ankle)	111	0.03*	1.04	0.69	1	0.04*	1.03
Hip (neck of femur fractures)	217	0.80	0.99	0.28	1	0.58	0.99
Periprosthetic	16	0.10	1.19	0.02*	0.99	0.12	1.16
Pelvis (acetabulum, pelvic ring)	25	0.20	1.1	0.22	1	0.16	1.1
Femur (shaft and distal femur)	18	0.31	1.09	0.75	1	0.24	1.09
Patella	6	0.55	0.93	0.92	1	0.61	0.94
Tibia	42	0.10	0.98	0.79	1	0.22	0.99
Spine	64	0.23	0.95	0.79	1	0.16	0.95
Rib fracture/chest trauma	38	0.79	0.99	0.75	1	0.88	0.99
Clavicle	25	0.20	1.11	0.62	1	0.15	1.11
Humerus	70	0.12	1.06	1.00	1	0.05	1.08
Forearm	60	0.52	1.004	0.99	1	0.67	1.003
Distal radius	90	0.87	1.006	0.75	1	0.94	1.003

Several meteorological factors (average temperature, total hours of sunlight, and dew point) were significantly correlated with the age group of presentation, mechanism of injury, or type of injury (Table 2).

The neck of femur fractures, which account for 27% of trauma pathology treated in Lister Hospital, were not significantly correlated with any meteorological factor studied. On the contrary, the second most common trauma pathology, ankle trauma, accounting for 15% of all local trauma, were the only injuries significantly correlating with temperature (p < 0.03) and dew point (p < 0.04) but not with the total amount of sunlight (p < 0.69). Lower temperatures and dew points were associated with ankle trauma.

Trauma sustained from the most common mechanism, a ground-level fall, was not significantly associated with any of the meteorological factors studied (temperature, sunlight, and dew point). From all four mechanisms of injury recorded, a fall from height significantly correlated with sunlight as the only meteorological variable (p < 0.04), and most falls from a height correlated with a lower total duration of sunlight.

With regards to the effect of weather and its effect on the age group of presentation, temperature (p < 0.01), sunlight (p < 0.002), and dew point (p < 0.03) were all significantly associated with trauma in patients aged younger than 21 years of age. The higher the temperature and dew point and longer the duration of sunlight, the more likely it was that trauma pathology presented in this age group.

The temperature was the only meteorological factor significantly correlated with trauma pathology presenting in the age group of 35-44 years (p < 0.03); the higher the temperature, the more commonly it was associated with trauma. There was no other correlation observed between the effect of the studied weather variable on this age group of presentation.

## Discussion

We demonstrated no significant correlation between weather and neck of femur fractures but noted a statistically significant relationship between adverse weather and ankle trauma. Our paper is a systematic review of the effect of weather on trauma workload and it highlights the importance of understanding the effect of specified weather conditions in different localities [1]. This paper quantified the effect of weather on a 180-day period on the trauma workload within our area, which is the first to address this geographical area in both full seasons of autumn and winter.

Our region lies at the benign end of the northwest-to-southeast climatic gradient across the British Isles, consisting of low rainfall, high sunshine, and high summer temperatures [10]. There is a tendency for the region to experience lengthy (> one year) periods of below-average rainfall. The meteorological factors studied were sunlight, temperature, and dew point. The dew point indicates the amount of moisture in the air, it is the temperature the air needs to be cooled to achieve a relative humidity of 100%, beyond this point, the air cannot hold more water in the gas form and thus water will come out of the atmosphere in liquid form as fog or precipitation making ground surfaces moist [11,12].

Our most common trauma presentations were the neck of femur fractures, which accounted for 27% of our injuries but they did not correlate with any weather variable; this is in line with Lofthus et al.'s hip fracture study, which showed there was no significant seasonal variation in the incidence of hip fractures with no correlation between the mean outdoor temperature and number of fractures for each month [13].

On the contrary, ankle injuries accounted for 15% of our local trauma and were significantly correlated with temperature and dew point, which indicated that the moister outdoor conditions are the more likely a patient is to slip and sustain an ankle injury whilst pursuing an outdoor activity such as running, walking, or cycling.

This local study confirmed that meteorological variables do affect trauma workload in patients aged younger than 21 years of age, who account for more than 20% of the population of Hertfordshire, with 71% of such injuries occurring outdoors in favourable meteorological weather variables, with patients aged 35-44 years more likely to sustain trauma in warmer environments [14-16].

Falls occur at work, at home, and in recreational places. Through analysing the mechanism of injury, unsurprisingly, a fall from height was the only mechanism found to be significantly associated with the duration of sunlight, with most falls occurring on days with the least duration of sunlight available. It may be hypothesised that secondary to the current popularity of do-it-yourself (DIY) home renovation projects, these incidents came as a result of attempting to finish these before dusk after a day's work [17,18].

Our institution saw the highest number of consultations and operations take place during the months of October and November, consistently featuring higher trends on Tuesdays and Fridays. During these time periods, clinical directors may experiment with having more trauma operating capacity and registrars around to support the acute trauma service to treat patients in a timely manner. Perhaps, staff should avoid taking time off during the months of October and November when the level of referrals and operating requirements are at their highest peaks. It was surprising that December demonstrated a large drop in the number of consultations with our department possibly related to the fact that many families travel during school holiday times.

Limitations of this study include the fact that the study period includes the months of autumn and winter only. Unfortunately, the study was brought to a halt due to the effect of another phenomenon beyond our control, which was the novel coronavirus. Despite that, this is the first study assessing the effects of meteorological factors on trauma in our area and it lacks generalisability to other areas with different microclimates. However, this echoes the need for further studies in different localities, which need to analyse their trauma requirements during the year to plan their trauma resources and staffing allocation.

## Conclusions

In light of our findings, we conclude that weather has no effect on the neck of femur fractures, the most common trauma pathology treated in our institution. Thus, in all seasons, allocated specific trauma lists should be arranged irrelevant of the weather conditions. Weather variations are significantly associated with ankle injuries, the second most common trauma pathology demonstrating that the more moist the atmosphere, the more susceptible one is to ankle trauma. We identified that Tuesdays and Fridays received the highest referral rate and peaked between the months of October and November. These data lay the groundwork for local clinical directors to shape the future on-call trauma service.
